# Inflammasomes in Common Immune-Related Skin Diseases

**DOI:** 10.3389/fimmu.2020.00882

**Published:** 2020-05-12

**Authors:** Lili Tang, Fusheng Zhou

**Affiliations:** ^1^Department of Dermatology, The First Affiliated Hospital, Anhui Medical University, Hefei, China; ^2^Institute of Dermatology, Anhui Medical University, Hefei, China; ^3^Key Laboratory of Dermatology (Anhui Medical University), Ministry of Education, Hefei, China; ^4^Inflammation and Immune Mediated Diseases Laboratory of Anhui Province, Hefei, China

**Keywords:** inflammasome, immune-related, psoriasis, vitiligo, systemic lupus erythematosus, atopic dermatitis, mouse model

## Abstract

The inflammasome is an important protein complex that cleaves the proinflammatory cytokines pro-IL-1β and pro-IL-18 into their active forms. Owing to its critical role in eliciting innate immune responses, IL-1β has been suggested to contribute to various skin diseases, including psoriasis, vitiligo, systemic lupus erythematosus (SLE), and atopic dermatitis (AD). Recently, several types of activators and inhibitors of different inflammasomes, as well as inflammasome-related genes and genetic susceptibility loci, have been identified in these immune-related common skin diseases. In particular, inflammasome activators and inhibitors presented highly cell-type-specific activity, suggesting that the inflammasome might perform different functions in different cell types. Moreover, most of these findings were based on experimental disease models, and the clinical features of the models partly resemble the typical symptoms of the diseases. In this review, from the perspective of activators and inhibitors, we collected evidence from the widely-studied inflammasomes, NLRP3, AIM2, and NLRP1, in psoriasis, vitiligo, SLE, and AD. Importantly, some small-molecule inhibitors hold therapeutic promise for the treatment of these diseases.

## Introduction

The human skin is composed of epidermal and dermal layers that function as the first line of defense against various physical, chemical, and biological threats. The major structure of the epidermis can be divided into four tightly-connected and stratified parts, namely, the stratum basale, stratum spinosum, stratum granulosum, and stratum corneum (1). Skin homeostasis is maintained by various stem cells, which are responsible for organ renewal and injury repair. The strong self-renewal activity of these cells results in different cell lineages that comprise the mature adult tissue (2). Keratinocytes are the main cell type found in the epidermal layer; however, human skin also contains different types of immune cells, such as memory αβ-T cells, dendritic cells (DCs), macrophages, natural killer (NK) cells, δγ-T cells, innate lymphoid cells (ILCs), and melanocytes (3, 4). These cell types coordinate cutaneous immune responses against external stimuli. Aberrant immunological activation by microorganisms or autoantigens can induce inflammatory skin disorders, and even cancers.

The inflammasome is a high-molecular-weight protein complex found mainly in the cytosol of stimulated immune cells, and plays an important role in activating immune cascades by processing and generating the catalytically active protease caspase-1 (5). Caspase-1 further initiates downstream responses through its substrates gasdermin-D, interleukin (IL)-1β, and IL-18 and induces a type of cell death called pyroptosis (6). The inflammasome is a sensor for monitoring extra- and intracellular compartments for signs of infection or tissue injury, and is therefore regarded as a key component of the innate immune system. When the inflammasome was first described by Martinon et al. (5), it was implicated in almost every immune-related phenotype, including tissue healing, metabolism, infection, homeostasis, and tumorigenesis (7). The past 15 years have seen great progress in deciphering the role of the inflammasome in the pathophysiology of common skin diseases. In this review, we mainly focus on two well-studied types of inflammasomes, NLRP3, and AIM2. For activation and regulation information of the various inflammasomes, please see the relevant reviews (8, 9). We also discuss the main findings of inflammasome-related genes in common immune-related skin diseases, including psoriasis, vitiligo, systemic lupus erythematosus (SLE), and atopic dermatitis (AD).

## Basic Concepts of the Inflammasome

**Figure 1:**
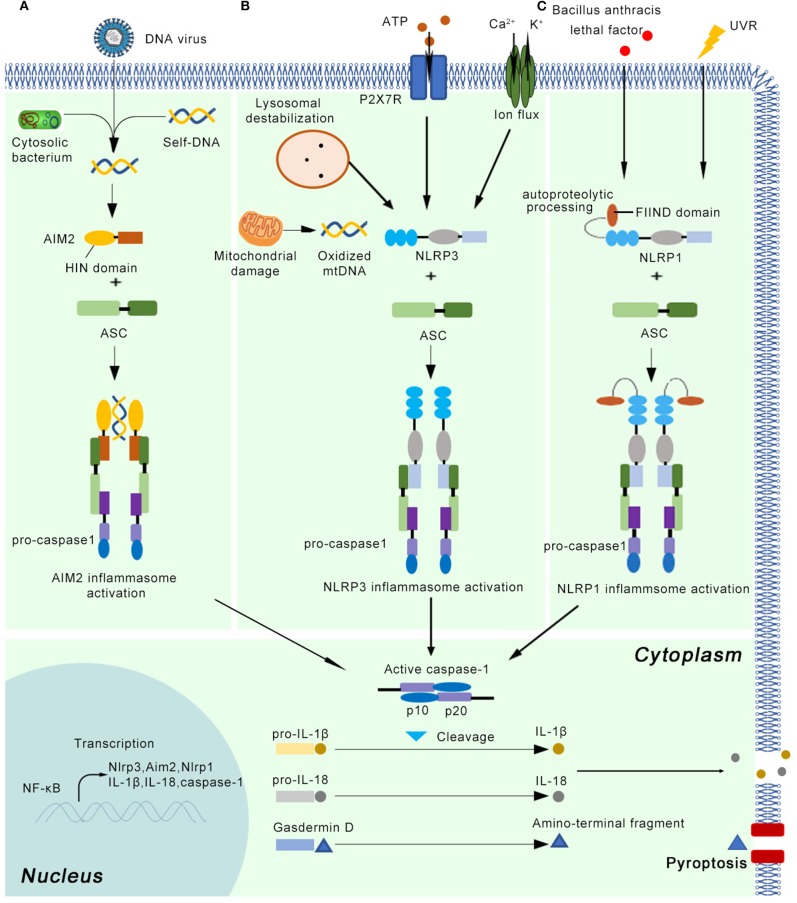
The basic concepts of the AIM2, NLRP3, and NLRP1 inflammasome. After priming by cytokine signals, the main components of the inflammasome (Nlrp3, Aim2, Nlrp1, pro-caspase-1, pro-IL-1β, and pro-IL-18) are transcribed in a NF-κB dependent manner. **(A)** The AIM2 inflammasome detects cytosolic dsDNA released from DNA viruses and cytosolic bacterium, as well as self-DNA. AIM2 senses and binds cytosolic dsDNA longer than 200 bp *via* its HIN domain. **(B)** The NLRP3 inflammasome can be activated by a variety of endogenous molecules, such as oxidized mitochondrial DNA, potassium efflux, extracellular ATP, lysosomal destabilization, intracellular calcium levels. **(C)** Autoproteolytic processing within the function-to-find domain (FIIND) is needed for the NLRP1 inflammasome activation. UV radiation and lethal factor of *Bacillus anthracis* can activate the NLRP1 inflammasome. Once the active inflammasome is formed, it directly recruits and cleaves pro-caspase1 into active caspase-1, which proteolytically activates the pro-inflammatory cytokines IL-1β and IL-18. In addition, the activated inflammasome cleaves gasdermin D into active N-terminal fragment, which drives a lytic type of cell death pyroptosis.

The inflammasome can recognize damage-associated molecular patterns (DAMPs) or uncontrolled release of pathogen-associated molecular patterns (PAMPs) through pattern recognition receptors (PRRs). At least five canonical and several non-canonical inflammasomes have been identified based on the type of PRR involved (9). The five canonical PRRs comprise nucleotide-binding oligomerization domain (NOD)-like receptors (NLRs), RIG-like receptors, absent in melanoma 2 (AIM2)-like receptors (ALRs), Toll-like receptors (TLRs), and pyrin. The canonical inflammasomes mainly activate the protease caspase-1, while non-canonical inflammasomes target caspase-11 in mice and caspase-4 and/or−5 in human cells (10, 11). In addition to the recognition receptors, canonical inflammasome assembly requires the apoptosis-associated speck-like protein containing a caspase recruitment domain (ASC) and effector protein pro-caspase-1 (12). The inflammasome must be tightly regulated through transcriptional, translational, and posttranslational mechanisms owing to its critical importance in innate immunity. First, the IL-1 receptor or other cytokines trigger the expression of inflammasome components (ASC, pattern receptors, pro-caspase-1, pro-IL-1β, and pro-IL-18). Second, DAMP- and PAMP-mediated signaling initiates the assembly of the multiprotein inflammasome, which entails pro-caspase-1 activation and cleavage of pro-IL-1β and pro-IL-18 into their active forms (13) (Figure 1). Secreted cytokines then induce effector cells, such as neutrophils, macrophages, and keratinocytes, to instigate inflammatory responses in damaged tissue (14).

### The NLRP3 Inflammasome

The NLRP3 inflammasome is the most complex and best-characterized member of the inflammasomes (5) (Figure 1). It can be primed by a wide range of extracellular inflammatory stimuli, such as bacteria, and viruses, as well as yeasts such as *Candida albicans* (15) and *Malassezia* spp. (16), in a NF-κB-independent manner (17). In addition, the NLRP3 inflammasome is activated in response to a variety of endogenous molecules indicative of tissue injury, such as oxidized mitochondrial DNA (18), potassium efflux (19), extracellular ATP (20), lysosomal destabilization (21), and intracellular calcium levels (22). The priming step results in the transcriptional induction of *NLRP3* and activation of licensing receptors. Importantly, NLRP3 inflammasome activation can also be controlled by kinases such as Bruton's tyrosine kinase and JNK or Syk kinases through the recruitment of caspase-1 and regulation of ASC oligomerization, respectively (23–25). In human monocytes and macrophages, adenosine triphosphate (ATP) stimulation through P2X7R is also required to activate the NLRP3 inflammasome (26). After priming, NLRP3 oligomerization mediates the cleavage of pro-caspase-1, pro-IL-1β, and pro-IL-18 into their active forms (27). Although numerous regulators have been identified in both the priming and oligomerization stages, the exact mechanism by which NLRP3 is activated remains unclear (28).

### The AIM2 Inflammasome

The AIM2 inflammasome contains AIM2 as the recognition receptor. AIM2 mainly detects cytosolic dsDNA released from viruses and intracellular bacteria, as well as self-DNA (29) (Figure 1). By inducing the expression of IRF1, the host system controls the expression of GTPases known as guanylate-binding proteins (GBPs), which facilitates the sensing of cytosolic dsDNA. AIM2 senses and binds cytosolic dsDNA longer than 200 bp *via* its HIN domain, providing an oligomerization template (30). However, the mechanisms underlying how external DNA is sensed by PPRs are normally species-dependent. For example, *Francisella novicida* infections activate the AIM2 inflammasome through the interferon-inducible proteins GBP2, GBP5, and IRGB10 (31, 32), whereas GBP1 is required for AIM2 inflammasome-mediated detection of *Salmonella* (33).

In addition to its role in detecting exogenous bacterial DNA, the AIM2 inflammasome has been suggested to monitor self-DNA delivered by exosomes or damaged DNA within the nucleus (34, 35). AIM2-deficient mice are protected from ionizing radiation-induced cell death and severe tissue damage, suggesting that AIM2 mediates inflammasome activation through sensing dsDNA damage induced by exposure to ionizing radiation (34). Treatment with the cytotoxic agent irinotecan (CPT-11) leads to considerable intestinal release of dsDNA through exosome secretion, which then enters into innate immune cells and triggers the AIM2 inflammasome-mediated secretion of mature IL-1β and IL-18 (35).

### The NLRP1 Inflammasome

NLRP1 is another member of NLR family that forms a new kind of inflammasome in human. NLRP1 inflammasome can mediate homotypic interactions through the PYD domain, using the same strategy as NLRP3 inflammasome. Interestingly, unlike NLRP3 protein, NLRP1 also has a function-to-find domain (FIIND) and a caspase activation and recruitment domain (CARD) (5) (Figure 1). NLRP1 inflammasome also interacts with ASC via CARD domain and activates their proteolytic function, which means this inflammasome can activate caspase-1 by CARD domain without recruiting ASC (36). Except for protein-protein interaction, additional event is needed for NLRP1 inflammasome activation. It has been showed that autoproteolytic processing within FIIND domain is necessary for the NLRP1 activity (37). UVB irradiation can activate NLRP1 inflammasome in human primary keratinocytes but not human fibroblasts (38). However, it is argued that NLRP3 inflammasome might also induce secretion of IL-1β and IL-18, indicating further investigation is needed (39). The lethal factor of *Bacillus anthracis* can activate NLRP1 inflammasome in rodents (40). The activation of NLRP1 inflammasome results in downstream response, including release of active caspase-1 cleavage enzyme, IL-1β and IL-18, as well as the activation of pyroptosis (41).

## Inflammasomes in Systemic Lupus Erythematosus

**Figure 2:**
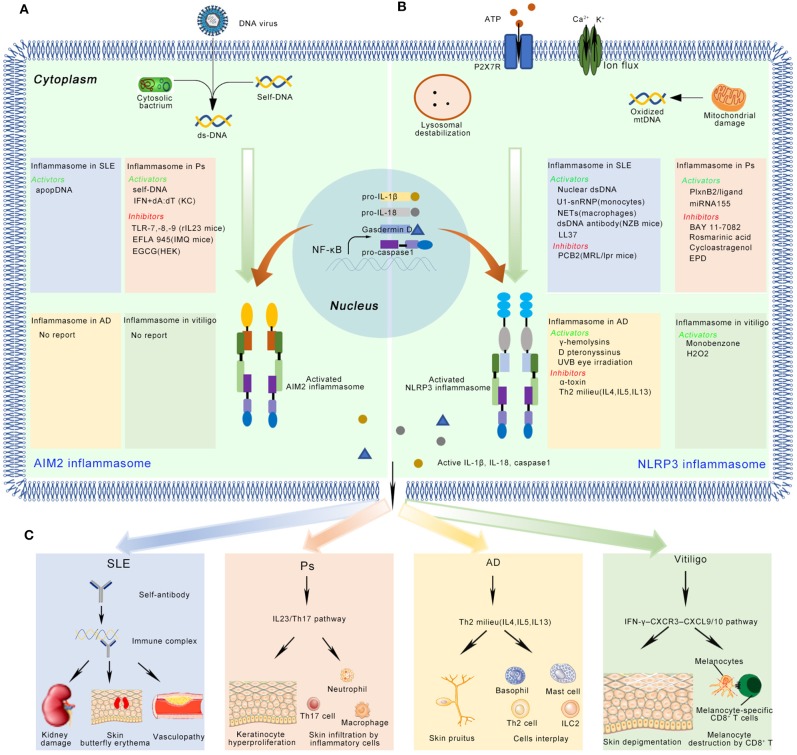
The activation and inhibition of the AIM2 and NLRP3 inflammasome in SLE, Ps, AD, and vitiligo. **(A)** In SLE, the AIM2 inflammasome can be activated by apopDNA in the patients. In psoriasis (Ps), the self-DNA in patients, dA:dT (after primed by IFN in keratinocyte) activate, while TLR-7/8/9, EFLA 945, and EGCG can inhibit the AIM2 inflammasome in Ps mice model or cultured cell lines. **(B)** In SLE, several NLRP3 activators (Nuclear dsDNA or dsDNA antibody, U1-snRNP, NETs, LL-37), and inhibitors (PCB2) have been identified in mice model or cell lines. In Ps, PlxnB2/ligand and miRNA155 activate the NLRP3 inflammasome, while BAY 11-7082, Rosmarinic acid, Cycloastragenol and EPD inhibit this inflammasome. In AD, activators (γ-hemolysins, D pteronyssinus, UVB eye irradiation) and inhibitors (α-toxin, Th2 milieu) of the NLRP3 inflammasome have been reported. In vitiligo, monobenzone, and H2O2 activate the NLRP3 inflammasome. **(C)** The active IL-1β and IL-18 might play multiple functions in immune skin disorders. Here, we showed their potential roles in the disease pathogenesis. In SLE, autoantibody binds with apopDNA, and generates immune complex. The immune complex might cause kidney damage, skin butterfly erythema and vasculopathy when it is deposited into different tissues. In Ps, IL-1β activates IL23/Th17 pathway, inducing a large number of inflammatory cytokines and chemokines. Neutrophils, Th17 cells, macrophages, and some other kind of immune cells infiltrate into skin. These immune cells crosstalk with keratinocytes and finally cause hyperproliferation in epidermis. In AD, the inflammasome regulates disease mainly through Th2 milieu. The typical symptoms include chronic, pruritic eczematous skin and the elevated serum concentrations of IgE. In vitiligo, the disease is regulated by IFN-γ-CXCR3–CXCL9/10 axis. The skin-resident melanocytes-specific CD8+ cells kill melanocytes, and finally cause depigmented skin patches.

SLE is a severe, devastating heterogeneous autoimmune disease characterized by loss of tolerance to self-antigens, chronic inflammation, and strong interindividual variation. The pathophysiology of SLE is highly complex and remains incompletely understood; however, there is substantial evidence indicating that the inflammasome might be involved in regulating cytokine secretion and inducing chronic inflammation in this condition, thereby partly contributing to disease development (Figure 2). Kahlenberg et al. were the first to observe that the expression of IL-1β/IL-18 was upregulated in the serum of SLE patients. They also found that inhibition of caspase-1 induced the aberrant differentiation of endothelial progenitor cells (EPCs) and circulating angiogenic cells (CACs), suggesting that the inflammasome machinery was involved in SLE etiology (42). In a clinical trial, IL-18 was found to be a predictive marker for long-term renal outcome. After 6 months of treatment, the serum IL-18 level in pediatric-onset SLE patients was significantly correlated with SLE global disease activity and the severity of lupus nephritis, suggesting that the NLRP3 inflammasome might modulate SLE treatment through IL-18 (43). A recent study revealed that *Casp1*^−/−^ mice were strongly protected against pristane-induced autoantibody development and type I interferon responses, indicating that caspase-1 is an essential component in lupus development (44).

### Genetic Evidence for Inflammasome Involvement in SLE

SLE has a strong genetic background, and more than a 100 susceptibility loci have been identified (45). Some of these loci are located near or within genes coding primarily for inflammasome components (Table 1). Two genetic association studies revealed that variations in *IL1B* and *NLRP1* are associated with SLE in Brazilian cohorts (47, 51). Several studies confirmed that genetic variation in the type I IFN signaling pathway increased the risk for developing SLE in humans and model mice (61, 62). Polymorphisms in two key receptors for NLRP3 priming, namely, P2X7R and TLR-9, are found to be associated with SLE in patients of different ethnicities (46, 48–50).

**Table 1:** Genetic evidence for inflammasome involvement in immune-related skin diseases.

**Gene**	**Variants**	**Sample size (case/control)**	**OR**	**Population**	**Disease subtype**	**References**
**SLE**						
*TLR7*	rs179008-T	282/309	1.74	European	SLE	(46)
*NLRP1*	rs2670660-G	144/158	2.06	Brazilian	SLE	(47)
*TLR9*	rs187084-?	285/305	2.23	Chinese Taiwan	SLE	(48)
*P2X7R*	rs1718119 -G	535/532	0.64	Chinese	LN	(49)
*TLR9*	rs35214-T	430/424	1.43	Chinese	SLE	(50)
*IL-1b*	rs1143629-G	90/144	1.95	Brazilian	Juvenile-onset SLE	(51)
**Psoriasis**						
*NLRP3*	rs10733113-G	741/1002	2.06	Chinese	Psoriasis	(52)
*CARD8*	rs2043211-?	741/1002	1.3	Chinese	Psoriasis	(52)
*NLRP1*	rs8079034-C	773/802	1.45	Swedish	Psoriasis	(53)
*AIM2*	rs2276405-A	11245/11177	0.83	Chinese	Psoriasis	(54)
**Vitiligo**						
*NALP1*	rs6502867-A	114 families	2.08	USA and UK	Vitiligo/Autoimmune disease	(55)
*NALP1*	rs1008588-A	26/61	na	Jordanian	Vitiligo	(56)
*NALP1*	Nine mutation block	114 families	3.7	USA and UK	Vitiligo/Autoimmune disease	(57)
**AD**						
*NOD1*	Insertion/deletion-32,656	600 asthma/1,194 control	6.3	German	Atopic and non-atopic diseases	(58)
*CARD15*	R702W	392/297	1.98	German	AD	(59)
*NALP12*	In9	392/297	1.28	German	AD	(59)
*NLRP1*	rs12150220-?	1260/732	0.54	Swedish	AD	(60)

*SLE, systemic lupus erythematosus; AD, atopic dermatitis; LN, lupus nephritis; OR, odd ratio*.

### The NLRP3 Inflammasome in SLE

Several studies have suggested that NLRP3 has an important role in SLE (Table 2). As the studies used more than one cell type and mouse model, various inflammasome aspects can be viewed from different directions. We subjectively divided these studies into three categories based on study design: studies mainly based on cultured cells; those mainly based on SLE-like mouse models; and those based on mice carrying specific gene deficiencies.

**Table 2:** The inflammasome in SLE.

**Activator**	**Implicated genetic component**	**Cell type**	**Effector signal**	**Mouse model**	**Main findings**	**References**
**The NLRP3 inflammasome**						
U1-snRNP and its antibody	NF-κB, *Casp1*	Monocytes	IL-1β	Na	Activation of the NLRP3 inflammasome depends on ROS and K+ efflux.	(63)
Self dsDNA	NF-κB	Monocytes	IL-1β	Na	dsDNA and its autoantibodies activate the NLRP3 inflammasome; ROS and K+ efflux regulate inflammasome activation; high levels of IL-1β increase Th17 cell responses.	(64)
LPS, ATP	*Ifna, Irf1, Casp1*	Monocytes	IL-1β	Na	After priming with IFN-α, ATP activates the NLRP3 inflammasome in an IRF1-dependent manner.	(65)
Antagonist of TLR7, 8, and 9	*IL6, Nos2, Cxcl10, Tnfrsf9, Fasl*	na	IL-1β	NZBW	Inhibits inflammatory pathways.	(66)
Anti-dsDNA antibodies	*Tlr4*	Monocytes/ macrophages	IL-1β, IL-17A	NZB × NZW	Activated the NLRP3 inflammasome in monocytes/macrophages; induces the production of mitochondrial ROS.	(67)
IFN-α	*Aim2, Asc, Casp1*	PBMCs, EPCs, CACs	IL-1β, IL-18	NZM2328	Exogenous IL-18 inhibits endothelial differentiation in control EPCs/CACs; IFN-α contributes to an elevated risk of cardiovascular disease through suppression of the IL-1β pathway.	(42)
Procyanidin B2	*Asc, Casp1*	na	IL-1β, IL-18	MRL/lpr	PCB2 suppresses lupus nephritis in MRL/lpr mice by inhibiting the NLRP3 inflammasome.	(68)
Dnase1L3 inhibition	*Dnase1l3, Hmgb1*	BMDMs, THP1, HEK	IL-1β	*Nlrp3*^−/−^, *Casp1*^−/−^	Dnase1L3 inhibition separates cytokine secretion from pyroptosis by targeting ASC.	(69)
Anti-dsDNA antibodies	*Dnase1l3*	Monocytes, Dendritic cells	na	Dnase1l3LacZ, C57BL/6, *Rag1*^−/−^, *Sting^−/−^, Myd88^−/−^*	Self-antigen is digested by circulating DNASE1L3, DNASE1L3 might be a modulator following NLRP3 inflammasome activation.	(70)
DC-Abca1/g1 deficiency	*Abca1, Abcg1*	Macrophages, T-cells, DCs	IL-1β, IL-18	*Abca1^−/−^, Abcg1^−/−^, Nlrp3^−/−^*	DC-Abca1/g1 deficiency enhances T cell activation, cholesterol accumulation, Th1 and Th17 cell polarization, and NLRP3 inflammasome activation.	(71)
Pristane	*Ifna, Tnf, Il1B*	Monocytes	IL-18	*Casp1^−/−^*	Caspase-1 might play roles in the cross-talk between environmental exposure and development of autoimmunity.	(44)
**The AIM2 inflammasome**						
ATP	*Card8*	Macrophages, PBMCs	IL-1β	Na	The AIM2 and NLRP3 inflammasomes might contribute sex-differentially to SLE pathogenesis.	(72)
IFN-α	*Stat1, Ifi202*	BMDMs, splenic T or B cells, RAW264.7, J774A.1	na	Na	Cell type and gender-dependent factors differentially regulate the expression of the AIM2 and p202 proteins.	(73)
Hormone E2	*Ifi202, Esr1*	Splenocytes, WT276, NIH 3T3	na	C57BL/6, B6.Nba2, NZB, *Esr^−/−^*	Sex hormones differentially regulate the expression of *Ifi202*.	(74)
p202	*Asc, Ifnb, Casp1*	BMDMs	IL-1β, IL-18	NZB, C57BL/6	Prevents AIM2-mediated ASC clustering.	(75)
Apoptotic DNA	*Ifna*	Macrophages, Fibrosarcoma, BMDMs	IL-1β	BALB/c	The AIM2 inflammasome is important for apopDNA-induced macrophage functional maturation and SLE.	(76)

*SLE, systemic lupus erythematosus; LPS, lipopolysaccharide; ATP, adenosine triphosphate; MCC950, a selective NLRP3 inhibitor; Oleuropein (OL), component of olive leaf extract; BMDMs, bone marrow-derived macrophages; EPCs, endothelial progenitor cells; CACs, circulating angiogenic cells; MDSCs, myeloid-derived suppressor cells; PBMCs, peripheral blood mononuclear cell; DCs, dendritic cells*.

#### Studies Based on Cultured Cells

Some monocyte-based studies have indicated the importance of the inflammasome in SLE. Nuclear dsDNA was shown to induce IL-1β secretion from human monocytes by activating the NLRP3 inflammasome, and this activation was modulated by reactive oxygen species (ROS) and K+ efflux. NLRP3 inflammasome activation led to increased IL-17 production from CD4+ T cells and triggered the downstream immune cascade (64). In addition to the endogenous DNA- and microbial nucleic acid-mediated inflammasome activation, endogenous U1-small nuclear ribonucleoprotein (U1-snRNP) was shown to activate the NLRP3 inflammasome in monocytes in the presence of anti-U1-snRNP antibodies (63). Type I interferons (IFNs) are important mediators of SLE. Two recent studies revealed that IFN-alpha levels are negatively correlated with the expression of NLRP3/NLRP1 inflammasomes. Exposure to IFN-alpha primed monocytes for inflammasome activation in an IFN regulatory factor 1 (IRF1)-dependent manner (65, 77).

#### Studies Based on SLE-Like Mouse Models

To date, several SLE-like mouse models have been developed, and while most partially mimic the clinical symptoms of this disease, none represent the entire spectra observed in SLE patients. Three types of spontaneous or induced models, NZB/NZM, MRL/lpr, and pristane-induced lupus, are widely used in inflammasome studies. Although these mice share some symptoms, they each present with specific clinical-like manifestations. NZB and NZM mice exhibit lymphadenopathy, anti-dsDNA IgG, and immune complex-mediated glomerulonephritis (78, 79), while MRL/lpr mice present with lymphadenopathy, DNA and RNA-directed autoantibodies, glomerulonephritis, and dermatitis (80). After pristane injection, the autoantibodies appeared in mice, along with the glomerulonephritis, arthritis, and anemia, and most of these phenotypes were type I interferon-mediated (81). Different types of inflammasome activators were identified using these models.

##### NZB and NZM mice

Antagonists of TLR-7,−8, and−9 inhibit NLRP3 inflammasome-related pathways in NZBW F1 lupus model mice, and therefore represent a potential therapeutic approach for lupus treatment (66). Furthermore, NZB mice exhibited an increase in IL-1β and IL-17A concentrations and the Th17/Treg cell ratio following injection of anti-dsDNA antibodies, suggesting that the NLRP3 inflammasome is involved in lupus pathogenesis in mice (67). Kahlenberg et al. found that neutrophil extracellular traps (NETs) can activate the NLRP3 inflammasome in lupus-affected macrophages, while LL-37 was shown to activate the NLRP3 inflammasome through P2X7 receptor-mediated potassium efflux (42).

##### MRL/lpr mice

Some SLE patients also displayed psychosis, seizures, and cognitive dysfunctions. MRL/lpr-derived strains have some advantages in the examination of these neuropsychiatric symptoms. MRL/lpr mice treated with procyanidin B2 (PCB2) showed reduced serum levels of IL-1β and IL-18 when compared with Nlrp3-deficient mice (68).

#### Studies Based on Mice Carrying Specific Gene Deficiencies

Dnase1L3 inhibition can block both NLRP3 and NLRC4 inflammasome-mediated secretion of IL-1β by targeting the ASC recruitment domain; however, it has little effect on NLRP3-dependent pyroptosis (69). *Dnase1l3*-deficient mice, a model of pediatric-onset SLE, showed the early presence of anti-dsDNA and anti-chromatin antibodies, suggesting that Dnase1L3 might function as a key modulator following NLRP3 inflammasome activation (70). Deletion of *Abca1/Abcg1* induces enlarged lymph nodes and enhanced Th1 cell polarization. In this SLE-like model, the NLRP3 inflammasome was activated in dendritic cells but not in macrophages or T cells. *NLRP3* deficiency significantly diminished the SLE-like symptoms, suggestive of the importance of regulating *Abca1/g1* in dendritic cells (71). A recent study revealed that *Casp1*^−/−^ mice are strongly protected against pristane-induced autoantibodies and type I IFN, indicating that caspase-1 is an essential component in lupus development (44).

## The AIM2 Inflammasome in SLE

### Studies Based on Cultured Cells

Accumulating evidence has suggested that the AIM2 inflammasome contributes to SLE pathogenesis (82) (Table 2). The levels of *AIM2* mRNA are upregulated in the liver, PBMCs, and spleen of SLE patients when compared with healthy individuals (83). *AIM2* mRNA expression is upregulated in macrophages derived from male, but not female, SLE patients after stimulation with 2 mM adenosine triphosphate (ATP), suggesting that the AIM2 inflammasome contributes to SLE in a gender-dependent manner (72). Similarly, Panchanathan et al. found that the expression of *AIM2* can be induced in bone marrow-derived macrophages (BMDMs), but not splenic T or B cells, indicating that the AIM2 inflammasome is activated in a cell-specific manner (73).

### Studies Based on Mouse Models

Recent studies have shown that p202 inhibits AIM2 inflammasome activation in response to cytosolic DNA (74, 75). IFN-inducible PYHIN and p202 are also associated with SLE (84). Macrophages derived from lupus nephritis-affected mice can be activated by apoptotic DNA (apopDNA). *AIM2* expression is closely correlated with macrophage activation, and inhibition of *AIM2* expression significantly ameliorates SLE syndrome in apopDNA-induced, lupus-affected mice (76).

## The Inflammasome in Psoriasis

Psoriasis is a common inflammatory skin disease characterized by red scaly papules and plaques. The key features of psoriasis include aberrant proliferation and differentiation of keratinocytes; excessive infiltration of immune cells, such as T cells and DCs, into the skin; and production of various inflammatory cytokines and chemokines (1). TNF, IL-23, and IL-17 are key cytokines for disease development. The antimicrobial peptide (AMP) LL-37 can aggregate with dsDNA and initiate a cutaneous self-amplifying autoimmune reaction (85). Skin keratinocytes are key proinflammatory cells that respond to harmful insults through the coordinated production of cytokines, chemokines, and AMPs (86). The crosstalk between infiltrated immune cells and keratinocytes is central to the IL-17-mediated inflammatory response in psoriasis (87).

The levels of key inflammasome components, including NLRC4, NOD2, CARD6, and IFI16, are elevated in psoriatic epidermis (88, 90). Recently, several studies have reported that the AIM2 inflammasome is an important component of skin innate immunity. Kopfnagel found that human keratinocytes express *AIM2* and respond to dsDNA with IL-1β secretion, indicating that the AIM2 inflammasome is a trigger for skin inflammation (91) (Figure 2).

### Genetic Evidence for Inflammasome Involvement in Psoriasis

*NLRP3* rs10733113 and *CARD8* rs2043211 were found to increase the risk of psoriasis in a Swedish population, supporting the hypothesis that inflammasome variation predisposes individuals to psoriasis (52). Similarly, there is some evidence that *NLRP1* rs8079034 also predisposes to psoriasis (53). Our group conducted a large-scale genome-wide association study in a Chinese population, and found that the rs2276405 *AIM2* coding variant significantly increased the genetic risk for psoriasis in AA allele carriers (54) (Table 1).

### The NLRP3 Inflammasome in Psoriasis

Although more than 40 psoriasis-like mouse models have been developed, imiquimod (IMQ)- and IL-23-induced models, or mice with specific gene deficiencies, are the most commonly used models in inflammasome studies (92). Various factors (microRNAs [miRNAs], genes, drugs) have been reported to activate or inhibit the NLRP3 inflammasome in cultured human keratinocytes or mice with IMQ-induced psoriasis (Table 3). A recent study reported that BAY 11-7082, an antagonist of NF-κB, can alleviate psoriasis-like dermatitis by inhibiting the NLRP3 inflammasome and the NF-κB pathway (97). PlxnB2 and its ligand were reported to activate inflammatory responses in keratinocytes through the NLRP3 inflammasome and the NF-κB pathway (94). In human primary keratinocytes, miRNA155 suppressor, rosmarinic acid (RA), cycloastragenol (CAG), and the effective part of *Datura metel* L. (EPD) have been shown to inhibit NLRP3-induced inflammatory cytokines such as IL-1β, IL-6, IL-8, CCL20, and TNF, indicating that they are therapeutic candidates for psoriasis treatment (89, 93, 95, 96). Different from that observed in the IMQ-induced psoriasis mouse model, recombinant IL (rIL)-23-induced psoriasiform dermatitis is largely dependent on the P2X7R signaling pathway. When *Nlrp3*-deficient mice were injected with rIL-23, psoriasiform phenotypes were considerably ameliorated, suggesting that the NLRP3 inflammasome contributes to this process. Diaz-Perez et al. further found that activation of the NLRP3 inflammasome occurred mainly through neutrophils and not keratinocytes or T cells (101). These findings supported that NLRP3 inflammasome activation might be primarily dependent on the source of activators and different mouse models used in these studies.

**Table 3:** The inflammasome in psoriasis.

**Activator**	**Implicated genetic component**	**Cell type**	**Effector signal**	**Mouse model**	**Main findings**	**References**
**The NLRP3 inflammasome**						
Rosmarinic acid, poly(I:C)	*Il6, Il8, Ccl20, Tnf*, NF-κB, *Casp1*	Keratinocytes	IL-1β	na	Rosmarinic acid markedly inhibits poly(I:C)-induced NLRP3 inflammasomes.	(93)
CD100-PlxnB2	*Casp1*	Keratinocytes	IL-1β	na	CD100 activates the NLRP3 inflammasome in keratinocytes through binding to PLXNB2.	(94)
miR-155	*Il4, Ifng, Tlr4*, NF-κB, *Nlrp3, Casp1*	Keratinocytes	IL-1β, IL18	BALB/c	miR-155 activates the NLRP3 inflammasome, but does not affect the TLR4/NF-κB signaling pathway.	(95)
EPD	*Il2, Il6, Il10, Il12, Il17, Il22, Il23, TNF, Mcpt1, Ifng, Tlr7, Tlr8, Traf6, Myd88, IKKA, IKBA*, NF-κB	Keratinocytes	IL-1β	IMQ-C57BL/6	EPD inhibits the production of imiquimod-induced inflammatory cytokines via the TLR7/8–MyD88–NF-κB–NLRP3 pathway.	(89)
CAG	*Tnf, Il6*	BMDMs, Dendritic cells, Neutrophils, T lymphocytes	IL-1β	IMQ-C57BL/6	CAG suppresses the assembly of the NLRP3 inflammasome complex.	(96)
IMQ, BAY 11-7082	NF-κB, *Stat3, Bcl2*, JNK, Il6, *Tnf, Il23*	na	IL-1β, IL-18	IMQ-C57BL/6, *NLRP3*^−/−^	BAY 11-7082 alleviates the dual NF-κB and NLRP3 inhibition-dependent psoriasis-like dermatitis.	(97)
IL-17, IL-22	*Cxcl1, Cxcl5, Il8, Defb1, Camp, S100a8, S100a9*, ROS	Keratinocytes	IL-1β	IMQ-C57BL/6, *Casp1^−/−^*	IL-17 and IL-22 enhance skin inflammation *via* the ROS-NLRP3-caspase-1 pathway.	(98)
IMQ, Ac-YVAD-CMK	*Casp1, 2*, and *4, Aim2, Il17, Il23, Il6*	Keratinocytes	IL-1β, IL-18	*LynDN^−/−^, Casp1^−/−^, Casp11^−/−^*	Caspase-1/-11 activation in immune cells induces psoriasis-like disease in mice.	(99)
IMQ	*Il1A, Il1B, IlR, Il23a, Il17A, Il22, S100a9, Krt6, Cxcl3, Myd88*,	na	Na	*IL-1α^−/−^, IL-1β^−/−^, IL-1α/β^−/−^, Nlrp3^−/−^, Asc^−/−^, Casp1*^−/−^, *Il1r1^−/−^, Myd88^−/−^*	IMQ-induced skin inflammation is independent on the NLRP3 inflammasome.	(100)
ATP, BzATP, POM1, A438079	na	Macrophages, Granulocytes, Neutrophils	IL-1β	*Nlrp3^−/−^, P2x7r^−/−^*, IMQ- and rIL-23-induced psoriasis model	P2X7R-induced inflammation is largely dependent on the IL-1β/NLRP3 inflammasome pathway and neutrophils.	(101)
**The AIM2 inflammasome**						
Cytosolic DNA, poly(dA:dT),	*Ifng, Tnf, Camp, S100a7, S100a15*	Keratinocytes	IL-1β	na	Cytosolic DNA triggers the activation of the AIM2 inflammasome and IL-1β in psoriasis; LL-37 blocks AIM2 inflammasome activation.	(102)
IFN-γ, IFN-α	*Il17, Il22, Il1a, Il4, Il13, Tnf*	Keratinocytes	Na	na	AIM2 is expressed in Langerhans cells and melanocytes in normal epidermis, but only in keratinocytes under inflammatory conditions.	(103)
EGCG, poly(dA:dT)	*Asc, Ifng*	HEKn	IL-1β	na	EGCG attenuates AIM2-induced IL-1β secretion by suppressing both IL-1β-mediated priming and poly(dA:dT)-induced ASC oligomerization.	(104)
EFLA 945	*Casp1, Asc, Il17*	Macrophages	IL-1β, IL-18	na	EFLA 945 attenuates IMQ-induced psoriasis-related proinflammatory responses.	(105)
Antagonist of TLR7, 8, and 9	*Defb4, S100a4, S100a7a, Camp*	na	IL-1β, IL-18	C57BL/6	Treatment with the antagonist reduces the expression of the inflammasome components NLRP3 and AIM2.	(106)
**The NLRP1 inflammasome**						
Isostearic acid	*Il1a, Csf, S100a8, S100a9, Il17a, Il23*	Neutrophils, Keratinocytes	IL-1β, IL-18	C57BL/6	Isostearic acid promotes NLRP1 inflammasome activation in cultured keratinocytes.	(107)

*IMQ, Imiquimod; Ac-YVAD-CMK, Caspase 1 inhibitor AcTyr-Val-Ala-Asp-chloromethylketone; BAY 11-7082, I-κB kinase-β antagonist; EPD, Datura metel L; CAG, Cycloastragenol; BzATP,2′(3′)-O-(4-benzoylbenzoyl) adenosine 5′-triphosphate; poly(dA:dT), polydeoxyadenylic acid-polydeoxythymidylic acid double-stranded homopolymer; EGCG, Epigallocatechin-3-Gallate; EFLA 945, Product of red grape vine leaf extracts*.

Although it is known that the inflammasome contributes to the pathogenesis of psoriasis, the role of caspases in disease development remains controversial. In IMQ-treated *Nlrp3*^−/−^ mice, psoriasiform lesions were comparable to those of normal controls; however, caspase-1 activity in the skin was markedly decreased, indicating that the NLRP3 inflammasome was required for caspase-1 activation, but dispensable for skin inflammation (100). However, Cho found that the NLRP3 inflammasome can be activated in IMQ-treated caspase-1-deficient mice, although the severity of psoriasis was much lower than in wild-type mice (98). Meanwhile, the functions of inflammation-related caspases were shown to be highly cell-type-specific. Activation of proinflammatory caspase-1 and caspase-11 in immune cells is sufficient to induce a psoriasis-like phenotype; however, in a psoriasis mouse model, these caspases are dispensable for inflammasome activation in keratinocytes/fibroblasts (99).

### The AIM2 Inflammasome in Psoriasis

Cytosolic DNA of psoriatic skin triggers inflammation through activation of the AIM2 inflammasome and IL-1β (Table 3). In cultured keratinocytes, however, transfection of poly (dA:dT) induced IL-1β secretion only after priming by interferon gamma, suggesting that a proinflammatory cytokine microenvironment is essential for AIM2 inflammasome activation (102). Koning showed that AIM2 is expressed exclusively in Langerhans and melanocyte cells in normal epidermis, but is significantly upregulated in keratinocytes under inflammatory conditions such as psoriasis, AD, and allergic contact dermatitis (103). In an rIL-23-induced psoriasis mouse model, a TLR-7,−8, and−9 antagonist inhibited the dermal expression of *Nlrp3* and *Aim2* and reduced the secretion of Th1 and Th17 cytokines in skin and serum, suggesting that inflammasomes might be a therapeutic target for psoriasis treatment (106). Because murine keratinocytes do not express pro-IL-1β, IL-18 is the only cytokine that can be cleaved into its active form by inflammasomes in these cells (114). Recently, Chung reported that red vine leaf extract (EFLA 945) greatly attenuated IMQ-induced psoriasis phenotypes by inhibiting the activity of the AIM2 inflammasome (105). Epigallocatechin-3-gallate (EGCG) has been shown to inhibit AIM2-induced inflammatory cytokines, and attenuate caspase-1 activation in interferon gamma-primed HEKn cells (104).

IL-18 receptor knockout mice treated with Aldara exhibited thicker epidermis than that seen in normal controls. Aldara, a type of cream composed mainly of isostearic acid and IMQ, can be used to induce psoriasis-like lesions. Walter et al. found that isostearic acid was the key component in activating the NLRP1 inflammasome in a mouse model, indicating that Aldara might stimulate psoriasis-like phenotypes in different immune pathways requiring both inflammasome and IMQ-induced response (107).

## The Inflammasome in Vitiligo

Vitiligo is an autoimmune skin disease characterized by the destruction of skin melanocytes and the presence of patchy white spots on the skin. Although the etiology of vitiligo has not been fully elucidated, evidence indicates that both genetic and environmental factors contribute to disease susceptibility (107). The IFN-γ-CXCR3–CXCL9/10 axis is suggested to be key for triggering skin inflammation by recruiting autoreactive CD8+ T cells (115) (Figure 2). Recently, Richmond et al. found that skin-resident memory CD8+ cells could not kill melanocytes by themselves, which should cooperate with recirculating memory CD8+ T cells so as to maintain the disease (116).

### Genetic Evidence for Inflammasome Involvement in Vitiligo

Relatively few studies have investigated the role of the inflammasome in melanocytes and/or vitiligo. Furthermore, most findings have come from genetic association studies (Table 1). In 2007, Jin et al. performed a genetic linkage, family-based association, target-region sequencing study on vitiligo-associated multiple autoimmune diseases, and found that single nucleotide polymorphisms (SNPs) near and within *NALP1* were associated with vitiligo in Caucasian patients (55). This was the first evidence indicating that the inflammasome had a role in vitiligo pathogenesis. The same research group identified that peripheral blood monocytes expressing the *NALP1* high-risk haplotype, covering L155H and M1184V substitutions, secreted a significantly greater amount of mature, bioactive IL-1β than those of other haplotype carriers. The high-risk haplotype did not lead to altered NALP1 mRNA or protein levels, indicating that this haplotype functions mainly through regulation of the NALP1 inflammasome (57). Consistent with their findings, *NALP1* variants were also reported to be associated with Jordanian Arab vitiligo patients (56).

### The NLRP3 Inflammasome in Vitiligo

In 2016, van den Boorn et al. found that monobenzone treatment resulted in melanocyte-specific skin inflammation characterized by macrophage infiltration and NK cell activation. Meanwhile, cutaneous lymph nodes showed an inflammasome-dependent influx of macrophages with a tissue-resident phenotype (108). However, recruitment of NK cells into the ear during monobenzone treatment was significantly inhibited in *Nlrp3*-deficient mice, suggesting that the NLRP3 inflammasome is key to monobenzone-induced inflammation in melanocytes. This indicates that the NLRP3 inflammasome and its downstream cytokines may be promising therapeutic targets for vitiligo treatment (108). Recently, Li et al. found that NLRP3 inflammasome activation was needed to promote innate immunity in keratinocytes. Deactivation of the NLRP3 inflammasome impaired CD8+ T cell recruitment and inhibited cytokine secretion in T cells derived from vitiligo patients (109) (Table 4).

**Table 4:** The inflammasome in vitiligo and AD.

**Activator**	**Implicated genetic component**	**Cell type**	**Effector signal**	**Mouse model**	**Main findings**	**References**
**The NLRP3 inflammasome in vitiligo**						
Monobenzone	*Asc*	Natural killer cells, Macrophages, Dendritic cells	na	*Rag2^−/−^, P2rx7^−/−^, Nlrp3^−/−^, Asc^−/−^, IL18^−/−^*	Monobenzone-induced memory natural killer cell formation is dependent on the NLRP3 inflammasome of macrophages.	(108)
H_2_O_2_	*Trpm2*, ROS, NF-κB, *Cxcl10, Cxcl16, Trpm2, Irf1, Asc, Casp1*	T cells, NHEK	na	na	Oxidative stress–induced NLRP3 inflammasome activation in keratinocytes promotes cutaneous T-cell responses in vitiligo.	(109)
**The NLRP3 inflammasome in AD**						
Staphylococcal alpha-toxin	*Il4, Il5, Il13, Il17, Il22, Ifng, Casp1, Asc*	Monocytes, Keratinocytes	IL-1β	Na	Impaired NLRP3 expression and function may be important for *Staphylococcus aureus*-induced chronic skin inflammation in AD.	(110)
*Dermatophagoides pteronyssinus*	*Asc, Casp1*, NF-κB, *Il8*	Keratinocytes	IL-1β, IL-18	Na	House dust mite allergens activate the NLRP3 inflammasome in the development of atopic dermatitis.	(111)
Ultraviolet B irradiation	*Il18bp, Tslp*	na	IL-18	NC/Nga	The NLRP3 inflammasome is implicated in the effects of UVB irradiation.	(112)
Hemolysins, Lipoproteins	*Nlrc4, P2rx7r, Asc, Lta, Casp1*	Macrophages	IL-1β	*Nlrp3^−/−^, Asc^−/−^, Casp1^−/−^, P2rx7r^−/−^, Trif^−/−^*	*S. aureus* hemolysins circumvent the requirement for ATP and the P2rx7 receptor to induce caspase-1 activation *via* the NLRP3 inflammasome.	(113)

*AD, atopic dermatitis*.

### Other Types of Inflammasomes in Vitiligo

Two studies reported that *NLRP1* levels were upregulated in both melanocytes and keratinocytes at the edge of progressing vitiligo lesions, which suggested that the NLRP1 inflammasome might drive the disease *via* two pathways (117, 118). In addition, NLRP1 and IL-1β levels in the skin may represent better markers than detection of lymphocyte infiltration to monitor vitiligo activity (117). There is some evidence to suggest that elevated IL-17 levels play important roles in stimulating inflammasome activation during vitiligo development (119, 120).

## The Inflammasome in Atopic Dermatitis

AD is a chronic inflammatory dermatosis characterized by pruritic eczematous skin lesions and increased serum concentrations of immunoglobulin E (IgE) (121). The levels of *AIM2* were found to be increased in keratinocytes derived from psoriasis and AD patients, which led to acute and chronic skin barrier disruption-related inflammation (103). Like most immune-related diseases, AD is affected by both genetic and environmental factors. The house dust mite and *Staphylococcus aureus* are common external agents that can trigger AD. Type 2 helper T cell-induced (Th-2 type) inflammation was reported to be essential for AD pathogenesis (122). IL-4, IL-13, and TSLP (thymic stromal lymphopoietin) are key players in Th-2 cell fate determination and in inducing the expression of IgE (1, 123). Meanwhile, skin barrier dysfunction is considered to be an important factor in AD etiology. For example, a nonsense mutation in the gene coding for the skin barrier protein filaggrin (FLG) can be observed in 20–40% of AD patients (124). Mutations in other genes such as *SPINK5* and *DSG1* that play roles in regulating stratum corneum formation or maintaining epithelial cell–cell tight junctions, also result in AD or AD-like dermatosis (125, 126).

Several studies have suggested that inflammasomes play key roles in disease development. In both human and mouse skin, the expression of IL-1A, IL-1B, IL-18, and IL-1RA was significantly higher in *FLG* mutant homozygous carriers than in either heterozygous carriers or wild-type subjects (127). AD-like dermatitis induced through *FLG*-deficiency was dependent on IL-1β and IL-1R1 signaling, but not NLRP3 inflammasome activation (128). Schuepbach-Mallepell found that the inflammasome inhibited the expression of *TSLP* and had a role in regulating Th1 and Th2 cell fate in the skin (129). In a chronic proliferative dermatitis animal model, *Nlrp3*- or *Casp1*- and -*11*-deficient mice showed reduced skin inflammation and delayed disease onset, suggesting that the inflammasome might be an important trigger for disease development (130).

### Genetic Evidence for Inflammasome Involvement in AD

Several studies have revealed the inflammasome-related genetic variation involved in AD pathogenesis (Table 1). The *NLRP1* coding variant rs12150220 showed a strong association with AD in a Swedish population. This SNP was located between the PYRIN and NACHT domains, potentially affecting inflammasome oligomerization (60). Polymorphisms in *NOD1* and *CARD15*, two components involved in inflammasome assembly, increased the risk for AD in different populations (58, 131). Recently, *NLRP3* polymorphisms have also been reported to be associated with AD (59, 132).

### The NLRP3 Inflammasome in AD

The expression levels of *NLRP3* and caspase-1 are lower in AD-affected skin than in healthy skin. Moreover, the gene expression of *NLRP3* and *ASC* was significantly reduced in human keratinocytes stimulated with Th2 cytokines (IL-4, IL-5, and IL-13) (110). Several inflammasome activators have been found to be triggers for skin inflammation in AD (Figure 2). However, the reported effects have been contradictory. For example, Munoz-Planillo et al. found that *S. aureus* gamma-hemolysins alone could activate NLRP3 inflammasomes in macrophage cells. Alpha- and beta-hemolysins could also trigger inflammasome activation when coupled with bacterial lipoproteins. Interestingly, this activation was not dependent on the P2X7 receptor or the TLR adaptor MyD88, suggesting that *S. aureus* hemolysins might circumvent the requirement for the P2X7 receptor to activate the NLRP3 inflammasome (113). Inconsistent with this finding, Niebuhr et al. found that the *S. aureus* exotoxin, alpha-toxin, inhibited NLRP3 inflammasome activity by suppressing the expression of *NLRP3, ASC*, caspase-1, and *IL1B* in keratinocytes. The same trend was found in monocytes treated with Th2 milieu (LTA+alpha-toxin+IL-4, IL-5, and IL-13), suggesting that NLRP3 inflammasome impairment might contribute to skin inflammation in AD (110) (Figure 2). *Dermatophagoides pteronyssinus* activated the NLRP3 inflammasome in keratinocytes, and the released proinflammatory cytokines, IL-1β and IL-18, exacerbated the AD-associated symptoms (111). UVB eye irradiation was reported to aggravate AD symptoms through the NLRP3 inflammasome (112) (Table 4).

## Conclusion

In the past 15 years, great progress has been made in discovering new inflammasome components and identifying new activators that trigger inflammation or stimulate the innate immune system in various organs, mouse models, and cell types. In this study, we reviewed the known canonical inflammasomes, especially the NLRP3 and AIM2 inflammasomes, and their roles in common immune-related skin diseases. After inflammasome-mediated cleavage, the proinflammatory cytokines IL-1β and IL-18 are processed into their active forms and further initiate downstream inflammation cascades, such as IL-23/Th17 signaling, in skin tissue.

For the important roles of IL-1β and IL-18 in immune-related skin disorders, several therapeutic drugs targeting of inflammasome components have been developed. For example, canakinumab, an anti-IL-1β monoclonal antibody, has been used to treat a generalized pustular psoriasis patient and resulted in complete remission of the lesions (133). P2X7 and EGCG have been shown to attenuate murine lupus symptoms by inhibiting the activation of NLRP3 inflammasome, thus can be viewed as a promising therapeutic agent in SLE treatment (134–136).

Several key points should be mentioned following this review. First, even though the inflammasome is essential for IL-1β and IL-18 cleavage, some inflammasome-independent mechanisms have been reported for IL-1β and IL-18 processing. For example, neutrophil- and macrophage-derived neutral serine proteinases, such as proteinase 3 (PR3) and cathepsin-G, can cleave pro-IL-1β into its bioactive form (137). Second, although at least 10 different types of inflammasome have been identified, most have not been extensively characterized (8, 9). It is not known whether they play roles in skin diseases similar to those played by AIM2 and NLRP3 inflammasomes, which requires further investigation. Third, activators and/or inflammasome types are highly cell-type specific. Inflammasome activators cannot initiate an immune response in all cell types, indicating that therapeutic inflammasome inhibitors, such as caspase-1/IL-1β inhibitors, may be effective in one cell type but not in others. This is very important in the clinical treatment of the relevant disease, because proper pharmacological inhibitors must be chosen that are likely to depend on the type of inflammatory infiltrate (138). Finally, because skin biology differs between humans and mice, caution must be exercised when translating experimental data from mouse models to humans. Although some inflammasome inhibitors show strong anti-inflammatory efficacy in mice, direct evidence for a similar effect in treating human skin diseases is still lacking.

## Author Contributions

FZ contributed to the conception and design of the paper, provided approval for publication of the content, and agreed to be accountable for all aspects of the work. LT drafted the work and revised it critically for the content.

## Conflict of Interest

The authors declare that the research was conducted in the absence of any commercial or financial relationships that could be construed as a potential conflict of interest.
